# A Systematic Review on Sleep Duration and Dyslipidemia in Adolescents: Understanding Inconsistencies

**DOI:** 10.5935/abc.20150121

**Published:** 2015-10

**Authors:** Gabriela de Azevedo Abreu, Laura Augusta Barufaldi, Katia Vergetti Bloch, Moyses Szklo

**Affiliations:** Universidade Federal do Rio de Janeiro, Rio de Janeiro, RJ – Brazil

**Keywords:** Sleep, Dyslipidemias, Adolescent, Review

## Introduction

Although many questions about the role of sleep remain unanswered, it is known that sleep is not only a physiological function, but also performs an important role in promoting growth, maturation and general health of children and adolescents^1^, contributing significantly to cognitive, emotional functions and school performance^2^. Currently, there is a tendency for the young population to have irregular sleeping hours, with differences in bed and wake-up times between weekdays and weekends, especially as they get older^2-4^.

There is a growing interest about the impact of sleep and its disorders on regulation of inflammatory processes and morbidities, particularly in the context of metabolic and cardiovascular diseases (CVD) and their complications^1^. In children and adolescents, cross-sectional^5-7^ and prospective^8,9^ studies have shown an association between overweight or obesity and few hours of sleep. In adults, there is evidence supporting this association, as well as correlations with insulin resistance, diabetes and cardiovascular diseases^10-15^.

Few hours of sleep can also play a role in the etiology of a key risk factor to CVD, dyslipidemia^12,14,15^. Physiologically, sleep reduction is associated with hormonal alterations that may promote the development of an atherogenic lipid profile, including increase of cortisol and ghrelin and reduction of leptin levels, in addition to sympathovagal responses^16-18^. In order to obtain more information about the association between lipid metabolism alterations and sleep duration specifically in adolescents, we have performed a systematic review of the literature.

## Methods

This systematic review was based on the guidelines of the Preferred Reporting Items for Systematic Reviews and Meta‑analyses (PRISMA) statement^19^.

The search was performed in the electronic databases Medline via Pubmed^20^ Lilacs^21^, Web of Science^22^, Scopus^23^ and Adolec^24^.

Selection of the descriptors used in the review process was made through MeSH (Pubmed’s Medical Subject Headings). The search was performed in English, using three concept blocks: the first with terms related to sleep (*sleep*); the second with terms related to adolescence (adoles*, teen*, student*, *youth*, *young*); and the third with terms related to lipids (lipid*, lipemia*, cholesterol, HDL, LDL, triglyceride*, lipoprotein*, hypercholesterolemia*, hypercholesteremia*, dyslipidemia*, dyslipoproteinemia*, hyperlipidemia*, hyperlipemia*, “high density lipoprotein cholesterol”, “low density lipoprotein cholesterol”). The Boolean operator “OR” was used for the combination of the descriptors within each block and the Boolean operator “AND” was used to combine the blocks amongst themselves. The truncation of terms was applied when necessary. No search limits were used for date, language, study design or sample size. The search was carried out in August 2014, contemplating articles published up to that date. Table 1 shows the search strategy used in each database.

**Table 1:** Search strategy used for each database

Pubmed	(sleep*[Title/Abstract] AND (adoles* OR teen* OR student* OR youth OR young[Title/Abstract]) AND (lipid* OR lipemia* OR cholesterol OR HDL OR LDL OR VLDL OR triglyceride* OR lipoprotein* OR hypercholesterolemia* OR hypercholesteremia* OR dyslipidemia* OR dyslipoproteinemia* OR hyperlipidemia* OR hyperlipemia* OR "high density lipoprotein cholesterol" OR "low density lipoprotein cholesterol"[Title/Abstract]))
Lilacs	sleep$ and (adoles$ OR teen$ OR student$ OR youth OR young) and (lipid$ OR lipemia$ OR cholesterol OR HDL OR LDL OR VLDL OR triglyceride$ OR lipoprotein$ OR hypercholesterolemia$ OR hypercholesteremia$ OR dyslipidemia$ OR dyslipoproteinemia$ OR hyperlipidemia$ OR hyperlipemia$ OR "high density lipoprotein cholesterol" OR "low density lipoprotein cholesterol")
Adolec	sleep$ [Words] and adoles$ OR teen$ OR student$ OR youth OR young [Words] and lipid$ OR lipemia$ OR cholesterol OR HDL OR LDL OR VLDL OR triglyceride$ OR lipoprotein$ OR hypercholesterolemia$ OR hypercholesteremia$ OR dyslipidemia$ OR dyslipoproteinemia$ OR hyperlipidemia$ OR hyperlipemia$ OR "high density lipoprotein cholesterol" OR "low density lipoprotein cholesterol" [Words]
Web of Science	(Topic(sleep*) AND Topic(adoles* OR teen* OR student* OR youth OR young) AND Topic(lipid* OR lipemia* OR cholesterol OR hdl OR ldl OR vldl OR triglyceride* OR lipoprotein* OR hypercholesterolemia* OR hypercholesteremia* OR dyslipidemia* OR dyslipoproteinemia* OR hyperlipidemia* OR hyperlipemia* OR "high density lipoprotein cholesterol" OR "low density lipoprotein cholesterol"))
Scopus	(TITLE-ABS-KEY(sleep*) AND TITLE-ABS-KEY(adoles* OR teen* OR student* OR youth OR young) AND TITLE-ABS-KEY(lipid* OR lipemia* OR cholesterol OR HDL OR LDL OR VLDL OR triglyceride* OR lipoprotein* OR hypercholesterolemia* OR hypercholesteremia* OR dyslipidemia* OR dyslipoproteinemia* OR hyperlipidemia* OR hyperlipemia* OR "high density lipoprotein cholesterol" OR "low density lipoprotein cholesterol"))

Criteria for article inclusion in the systematic review were as follows: (a) studies on adolescents older than 10 years old; (b) studies that evaluated the association between sleep duration in hours and any lipid marker; (c) original research article. Articles evaluating any kind of sleep-related disorder, review studies, and experimental studies with animals were excluded. It was decided not to include theses, dissertations, and monographs. We reviewed the bibliographic references of reviews, systematic reviews, and meta-analyses that were found in the databases.

The articles were selected by two epidemiologists (GAA and LAB), initially based on title reading and then on abstract reading. Of the selected abstracts, the full articles were reviewed. In case of disagreement between the two reviewers with regard to the inclusion criteria, the title, and the abstract or the full article was maintained to be further evaluated. In case of disagreement with regard to the inclusion criteria, a third person was consulted.

Data from included articles were extracted independently, in duplicate (GAA and LAB), using a standard form. After extraction, data were compared and discussed. We extracted information about authorship, publication date, study place, population study, type of study, methods of sleep duration measurement and lipid profile assessment, sleep duration in hours, lipid markers, measure of association used to evaluate the correlation between hours of sleep and lipid profile, and variables used for adjustment of regression models.

We used an adaptation of the Newcastle-Ottawa (NOS) Quality Assessment Scale for Case-Control and Cohort Studies^25^, from the Ottawa Hospital Research Institute, to assess the quality of the longitudinal study included in this review. We also used the same scale adapted by Flynn et al^26^ to assess the quality of cross-sectional studies.

Due to the great amount of methodological heterogeneity observed between the assessed studies, a narrative approach to synthesize the results of studies included in the present systematic review was considered a better strategy.

## Results

**Figure 1:**
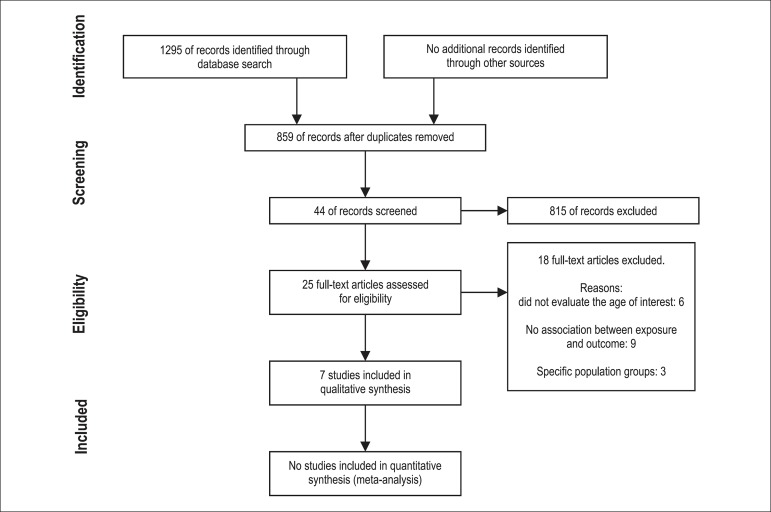
Flowchart of article selection.

The flowchart showing the selection process is shown in Figure 1. By the end of the evaluation process, of the 859 articles chosen after the removal of duplicates, 25 were submitted to full evaluation. Seven articles met the inclusion criteria at the end of the process.

Table 2 shows the relevant characteristics of the selected studies. Of the seven studies included, only one^27^ is longitudinal. The other six studies are cross-sectional. Five of the 7 studies^27-31^ included students. Sample sizes varied considerably, from 699 in the study by Rey-López et al^30^ to 14,267 adolescents in the study by Gangwisch et al^27^.

**Table 2:** Main characteristics of the selected studies

Reference/ Country	Study design/ Collection date	Study population	Age	Method for obtaining hours of sleep	Exposure classification (hours of sleep)	Method for lipid profile evaluation	Outcome (alterations of lipids)
Gangwisch et al.^27^, 2010/ United States	Longitudinal	Students, with national representativeness n = 14,257	11-21 years boys ≅ 15.8 years old	Questionnaire	Continuous	Questionnaire/ "Has any doctor ever (*between the 1st and the 3rd wave*) said you have high cholesterol?"	Dichotomous variable
	Wave I: 1994-95						
	Wave II: 1996						
	Wave III 2001-02	48.7% male	girls ≅ 15.9 years old				Yes/No
Kong et al^28^, 2011/ Hong Kong	Cross-sectional/ February 2007 -April 2008	Students* n = 1,274	12-20 years old†	Questionnaire^7^	<6.5h: 20%	Blood collection (TC, TG, HDL, LDL cholesterol)	Hypercholesterolemia
							TC ≥ 5.2 mmol/L
							LDL ≥ 2.6 mmol/L
				Actigraphy in sub-sample (n = 138)	6.5-8h: 40%		HDL <1.0 mmol/L
					>8h: 20%	Comparison of extreme quintiles	TG ≥ 1.7 mmol/L
Narang et al^29^, 2012/ Canada	Cross-sectional/ 2009-2010	Student n = 3,372 48.9% male	≅ 14.6 years old‡	Questionnaire^37,38^	Continuous	Capillary blood collection without fasting (TC and HDL cholesterol)	TC
							Borderline: 4.4-5.1 mmol/L:
							High: ≥ 5.2 mmol/L
					Quartiles		Non-HDL-cholesterol§
							Borderline: > 3.10 to 3.75 mmol/L
							High: > 3.75 mmol/L
Azadbakht et al^31^, 2013/ Iran	Cross-sectional Data from CASPIAN III//	Students n = 5,528	≅ 14.69 (2.45) years old boys	Questionnaire	< 5h	Blood collection (TC, TG and LDL)	Abnormal serum lipids were defined as TC, LDL-C and or TG higher than the level corresponding to the age and gender-specific 95th percentile^39^
			≅ 14.7 (2.38) years old girls		5 to 8h		
					> 8h		
Berentzen et al^32^, 2014/ Netherlands	Cross-sectional	General population n = 1,481	Mean age at completion of the questionnaire 11.4 (± 0.3) years	Questionnaire	7.5-9.5 h	Blood collection (TC and HDL cholesterol)	Continuous variable (mM)
		49% male	Mean age at the moment of medical examination 12.7 (± 0.4) years		10-10.5 h (ref. cat.)		
					11-12.5 h		
Rey-Lopez et al^30^, 2014/ Greece, Germany, Belgium, France, Hungary, Italy, Sweden, Austria, Spain	Cross-sectional/ 2006-2007	Students n = 699	≅ 14.8 years old	Questionnaire	Continuous variable	Blood collection (TG, TC and HDL cholesterol)	Continuous variable (mg/dL)
		52% male					
Lee et al, 2014/ Republic of Korea^33^	Cross-sectional/ 2007-2008	General population n = 1,187	≅ 15 years old	Questionnaire	≤ 5h	Blood collection (TG and HDL cholesterol)	Continuous variable (mg/dL)
		53% male			6-7h		
					8-9h (ref. cat.)		
					≥ 10h		

LDL: Low-density lipoprotein; HDL: High-density lipoprotein; TC: Total cholesterol; TG: Triglycerides; BMI: Body mass index.

* number of adolescents evaluated; total number of individuals evaluated in the study is 2,053, including children and adolescents;

† does not provide average age data or distribution by gender only for the adolescents’ group;

‡ does not provide age group;

§ non-HDL cholesterol corresponds to total cholesterol minus HDL cholesterol;

// CASPIAN III – Childhood and Adolescence Surveillance and Prevention of Adult Non-communicable disease.

All studies used questionnaires to obtain hours of sleep. The variable “sleep duration” was used as continuous in three studies^27,29,30^; whereas the other studies used different categories to classify sleep duration.

To obtain the lipid profiles, five studies collected venous blood^28,30-33^, one collected capillary blood^29^, and another used self-reported information^27^. Five studies measured total cholesterol^28-32^ and HDL-cholesterol^28-30,32,33^, four measured triglycerides^28,30,31,33^, and two evaluated LDL-cholesterol^28,31^. Almost all studies controlled for gender^27,28,30,33^ and age^27,28,30-33^; waist perimeter was adjusted for in two^28,29^, physical activity in four^27,30,31,33^, Tanner stage in two^28,32^, maternal level of education in two^31,32^, socioeconomic status in two^30,31^, body mass index (BMI) in one^28^, and caloric intake in one^33^.

The methodological quality assessment of the seven included studies is shown in Table 3. Only two cross-sectional studies^28,31^ obtained four points out of six in the bias risk evaluation. The longitudinal study showed a moderate risk of bias^27^.

**Table 3:** Evaluation of the risk of bias of the studies included

Study	Kong et al^28^, 2011/ Hong Kong	Narang et al^29^, 2012/ Canada	Azadbakht et al^31^, 2013/ Iran	Berentzen et al^32^, 2014/ Netherlands	Rey-López et al^30^, 2014/ Greece, Germany, Belgium, France, Hungary, Italy, Sweden, Austria, Spain	Lee et al^33^, 2014/ Republic of Korea	Gangwisch et al^27^, 2010/ United States
Sample representativeness	0	0	1	0	0	1	0
Definition of presenting condition	1	1	1	1	1	1	0
Evaluation of exposure	1	0	0	0	0	0	0
Evaluation of outcome	2	1	2	2	2	2	0
Nonresponse rate	0	0	0	0	0	0	0
Representativeness of the exposed cohort	0	0	0	0	0	0	1
Demonstration that outcome of interest was not present at start of study	0	0	0	0	0	0	0
Comparability of cohorts	0	0	0	0	0	0	1
Assessment of outcome	0	0	0	0	0	0	0
Was follow-up long enough for outcomes to occur	0	0	0	0	0	0	1
Adequacy of follow up of cohorts	0	0	0	0	0	0	1
Total	4/6	2/6	4/6	3/6	3/6	3/6	4/9

Cross-sectional studies (maximum 6 points) Sample representativeness: yes (1); no (0); not informed (0) Definition of presenting condition: classification based on two or more lipid markers (1); on only one lipid marker (0) Evaluation of Exposure (hours of sleep): combination of questionnaire with another evaluation method (1); only questionnaire (0) Evaluation of Outcome (lipid profile): venous blood (2); capillary blood (1); self-referred (0) Nonresponse rate: non-respondents described (1); non-described (0)

Cohort studies (maximum 9 points) Evaluation of Exposure (hours of sleep): combination of questionnaire with another evaluation method (1); only questionnaire (0) Evaluation of Outcome (lipid profile): venous blood (2); capillary blood (1); self-reported (0) Representativeness of the exposed cohort (representative of the average): adequately addressed (1); not adequately addressed ⁄ not reported (0) Demonstration that outcome of interest was not present at start of study: adequately addressed (1); not adequately addressed ⁄ not reported (0) Comparability of cohorts on the basis of the design or analysis: adequately addressed (1); not adequately addressed ⁄ not reported (0) Assessment of outcome (independent blind assessment or record linkage): adequately addressed (1); not adequately addressed ⁄ not reported (0) Was follow-up long enough for outcomes to occur: adequately addressed (1); not adequately addressed ⁄ not reported (0) Adequacy of follow up of cohorts (complete follow up or subject slost to follow up unlikely to introduce bias): adequately addressed (1); not adequately addressed ⁄ not reported (0)

Table 4 shows the main results of the associations found and the control variables each study used. Considering the seven studies included, only in three an association was found between hours of sleep and lipid profile^27,28,33^. Two studies found that shorter sleep duration was associated with a worse lipid profile (total cholesterol and LDL-cholesterol)^27,28^, and the results of the third one^33^ showed that long sleep duration was associated with high triglyceride levels. The other four studies^29-32^ did not find any association.

**Table 4:** Main results of the studies included in the review

		Total	Male		Female	Control variables investigated	
**Total cholesterol**							
Gangwisch et al^27^, 2010	OR (CI 95%)		OR (CI 95%)		OR (CI 95%)		Age/ gender/ race/ ethnic group/ alcohol/ smoke/ physical activity/ inactivity/ stress/ body weight
			Each hour: 0.91		Each hour: 0.85		
	Each hour: 0.87 (0.79-0.96)		(0.79-1.05)		(0.75-0.96)		
Kong et al^28^, 2011	β* = -0.160		---		---		Age/ sex/ BMI/ waist perimeter/ Tanner stages (2-3 and 4-5)
	(p-value = 0.023)						
Azadbakht et al^31^, 2013	---		OR (CI 95%)		OR (CI 95%)		Age/ socioeconomic status/ parents' level of education/ family history of chronic disease/ sedentary lifestyle/ BMI
			<5h = 1		< 5h = 1		
			5–8h = 4.00 (0.54–29.94)		5–8h = 1.07 (0.31–3.73)		
			> 8h = 5.63 (0.76–41.56)		>8h = 1.14 (0.33–3.85)		
Berentzen et al^32^, 2014	---		β (CI 95%)		β (CI 95%)		Age at completion of the questionnaire/ age at medical examination/ height/ maternal level of education/ puberty and screen time
			7.5–9.5 h = -0.15		7.5–9.5 h = -0.01		
			(-0.35; 0.04)		(-0.22; 0.21)		
			10–10.5 h =1		10–10.5 h = 1		
			11–12.5 h = -0.06		11–12.5 h = -0.06		
			(-0.17; 0.05)		(-0.16; 0.05)		
**LDL cholesterol**							
Kong et al^28^, 2011		β* = -0.122	---	---			
		(p-value = 0.042)					
Azadbakht et al^31^, 2013		---	OR (95%CI)	OR (95%CI)			
			< 5 h = 1	< 5 h = 1			
			5–8 h = 1.04 (0.30-3.61)	5–8 h = 1.36 (0.26–5.05)			
			>8 h = 0.97 (0.28–3.30)	>8 h = 0.76 (0.20–2.89)			
**HDL cholesterol**							
Kong et al^28^, 201		β* = -0.056	---	---			
		(p-value = 0.061)					
Berentzen et al^31^, 2014		---	β (95% CI)	β(95% CI)			
			7.5–9.5 h = 0.03	7.5–9.5 h = 0.07			
			(-0.07; 0.12) 1	(-0.03; 0.17)			
			10–10.5 h = 1	10–10.5 h = 1			
			11–12.5 h = 0.02	11–12.5 h = <0.01			
			(-0.04; 0.07)	(-0.05; 0.05)			
Lee et al^33^, 2014		OR (95%CI)		---			
		≤ 5 h = 0.79 (0.40 - 1.53)					
		6-7 h = 0.86 (0.50 - 1.49)	---				
		8-9 h = 1					
		≥ 10 h = 1.03 (0.44 - 2.40)					
**TG**							
Kong et al^28^, 2011		β* = 0.060	---	---			
		(p = 0.115)					
Azadbakht et al^31^, 2013		---	OR (95%CI)	OR (95%CI)			
			< 5 h = 1	< 5 h = 1			
			5–8 h = 1.09 (0.41–2.92)	5–8 h = 0.53 (0.22–1.30)			
			> 8 h = 1.16 (0.44–3.09)	> 8 h = 0.53 (0.22–1.30)			
Rey-López et al^30^, 2014	β (95%CI)		---		---		Age/ gender/ socioeconomic status/ physical activity
	School days: 0.26						
	(-2.57; 3.09)						
	Weekends: 0.69						
	(-1.50; 2.88)						
Lee et al^33^, 2014	OR (95%CI)		---		---		Age/ gender/ household income/ caloric intake/ physical activity
	≤ 5 h= 1.05 (0.55 - 2.00)						
	6-7 h= 1.20 (0.79 - 1.83)						
	8-9 h= 1						
	≥ 10 h = 2.17 (1.14 - 4.13)						
**Non-HDL†**							
Narang et al^29^, 2012	OR (95%CI)		---		---		Waist perimeter/nutrition/physical activity/sex/ family history of premature cardiovascular disease in first degree relatives/sleep disturbance score
	Each hour						
	1.03 (0.93-1.13)						
	First quartile (reference) x last quartile						
	0.92 (0.70-1.22)						
**TC/HDL-c**							
Rey-López et al^30^, 2014		β (95%CI)	---	---			
		School days: -0.001 (-0.05; 0.05)					
		Weekends: 0.009 (-0.03; 0.05)					
Berentzen et al^31^, 2014		---	β(95% CI)	β(95% CI)			
			7.5–9.5 h = -0.22 (-0.51; 0.08)	7.5–9.5 h = -0.18 (-0.44; 0.08)			
			10–10.5 h = 1	10–10.5 h = 1			
			11–12.5 h = -0.14 (-0.31; 0.02)	11–12.5 h = -0.04 (-0.17; 0.09)			

OR: OPdds ratio; CI: Confidence interval; SD: Standard deviation; LDL: Low-density lipoprotein; HDL: Gigh-density lipoprotein; TC: Total cholesterol; TG: Triglycerides; BMI: Body mass index; PR: Prevalence ratio.

* β regression coefficient of the multiple regression model to compare groups with the largest and smallest (reference) quintile of the lipid variables in relation to hours of sleep (group with 20% of individual with 20% of individual with longer sleep duration.);

† non-HDL cholesterol corresponds to total cholesterol minus HDL cholesterol.

In four studies^27,29,31,33^ the odds ratio was reported, whereas the other studies reported^28,30,32^ β coefficients from regression analysis.

## Discussion

The present systematic review showed lack of consistent evidence regarding the association between sleep duration and lipid profile in adolescents. Few studies were found and some had methodological limitations. There was great heterogeneity regarding the classification and type of analysis of sleep duration and lipid metabolism markers, which probably contributed to the inconsistency of the observed results.

Concerning heterogeneity between studies, this systematic review included studies that evaluated the outcome using different methods (self-reported^27^, capillary blood sample^29^, venous blood sample^28,30-33^) or with different interval duration between the measure of exposition and the outcome^32^.

Gangwisch et al^27^ did not exclude adolescents with dyslipidemia at baseline, thus, the incidence of dyslipidemia in adolescents could not be ascertained. Moreover, as the outcome established was self-reported, and the diagnosis of dyslipidemia depends on access to medical care, a bias may have occurred if adolescents from different socioeconomic status have different sleep habits.

All studies included in this systematic review obtained information about sleep duration based on questionnaires, a method frequently used in sleep research because of its easy application and low cost. However, the validity of the information obtained through questionnaires is of concern, particularly when the tools have not been submitted to a validation process. Adolescents may report only socially desirable sleeping and waking up hours^34^. Although all studies used questionnaires, sleep duration evaluation was also heterogeneous: one study asked the parents about the adolescent´s sleep duration^31^, one used pre-defined categories of bedtime and waking-up time^32^, while the others asked about sleep duration in an open question^27-30,33^.

Actigraphy – based on monitoring of activities – has been established as a valid and reliable method to evaluate sleep-wake patterns in children, adolescents and adults^35,36^. Objective methods for hours of sleep quantification in a population-based study are difficult to use, particularly in studies with relatively large samples. Kong et al^28^ used actigraphy in only about 7% of their study sample (138 out of 2,053) and demonstrated a reasonable agreement between actigraphy and adolescents’ self-reports (intra-class correlation coefficient = 0.72, CI 95%: 0.61-0.80).

In the studies included in this review, duration of sleep was measured in two different ways, as a continuous^27,29,30^ or categorical variable^28,31-33^. The lack of consensus about the best cut-off point to define short sleep duration makes it difficult to compare different studies, which would become easier if sleep duration were used as a continuous variable.

The present systematic review included a longitudinal study with important limitations and the cross-sectional studies showed associations in different directions. It was not possible to evaluate publication bias, due to the small number of studies identified. In summary, it is still uncertain whether there is an association between hours of sleep and lipid profile in adolescents. Heterogeneity regarding the way sleep hours were classified and analyzed, as well as the use of different lipids analytes may have contributed for the inconsistency of findings. More studies should be conducted on this issue to clarify the nature of this association and the involved biological mechanisms. These future studies must be longitudinal, use sleep duration as a continuous variable and consider the role of potential confounders or effect modifiers. Care must be taken to avoid over-adjustment, including variables that can be intermediary in the association between sleep duration and dyslipidemia such as BMI and food consumption.

Because of its strong association with cardiovascular disease in adults, it is important to identify and modify factors that are associated with lipid profile^15^ in adolescents. If short sleep duration is responsible for an unfavorable lipid profile, interventions that improve the quality and duration of sleep may contribute to decrease long-term cardiovascular risk.
